# Decrease in Resting Heart Rate Measured Using Smartphone Apps to Verify Abstinence From Smoking: An Exploratory Study

**DOI:** 10.1093/ntr/ntaa021

**Published:** 2020-01-23

**Authors:** Aleksandra Herbec, Ella Parker, Harveen Kaur Ubhi, Tobias Raupach, Robert West

**Affiliations:** 1 Department of Behavioural Science and Health, University College London, London, UK; 2 The Centre for Behaviour Change, Department of Clinical, Educational and Health Psychology, University College London, London, UK; 3 UCL Tobacco and Alcohol Research Group, University College London, London, UK; 4 Department of Clinical, Educational and Health Psychology, University College London, London, UK; 5 Department of Cardiology and Pneumology, University Medical Centre Göttingen, Göttingen, Germany

## Abstract

**Introduction:**

Verifying self-reports of smoking abstinence is challenging in studies that involve remote data collection. Resting heart rate (HR) decreases during smoking abstinence. This study assessed whether a decrease in resting HR measured using freely available smartphone apps could potentially be used to verify smoking abstinence.

**Methods:**

This study involved a repeated measures experimental design, with data collection in natural setting. Participants were 18 adult, daily smokers. They recorded resting HR in beats per minute (bpm) using freely available smartphone apps during five timepoints (two in the morning and three postnoon) on each of 3 days. The outcome measure was the mean of the postnoon HR recordings. The experimental condition for each of the 3 days (counterbalanced order) was as follows: (1) smoking as usual, (2) not smoking without nicotine replacement therapy (NRT), or (3) not smoking but using NRT. Abstinence was verified using expired-air carbon monoxide (CO) concentration.

**Results:**

Compared with the smoking as usual condition, mean HR was 13.4 bpm lower (95% confidence interval [CI] = 5.4 to 21.4, *p* = .001) in the not smoking without NRT condition and 10.4 bpm lower (95% CI = 3.1 to 17.8, *p* = 0.004) in the not smoking with NRT condition. There was no statistically significant difference in HR between the two not smoking conditions (*p* = .39). Abstinence during not smoking days without and with NRT was CO-verified in 18/18 and in 16/18 cases, respectively.

**Conclusions:**

Self-recording of resting HR in natural setting using smartphone apps shows a reliable decrease in response to smoking abstinence and may provide a basis for remote verification in smoking cessation studies.

**Implications:**

Remote verification of self-reported abstinence in smoking cessation studies remains challenging. Smoking abstinence has been shown to decrease resting HR under laboratory conditions. This study demonstrated that self-recording using freely available smartphone apps shows reliable decreases in resting HR during smoking abstinence and may provide a basis for inexpensive remote verification of smoking abstinence.

## Introduction

In studies of smoking cessation, it is important to be able to verify self-reports of abstinence.^1^ In studies of remote interventions, such as stop smoking websites and smartphone apps, this is particularly challenging. It has long been known that resting heart rate (HR) declines substantially within a day of stopping smoking and the decrease persists indefinitely.^2–5^ Most smartphones can run free apps that use the phone’s camera to assess the changes in the color of the fingertip with each heartbeat to measure HR.^6–8^ The current study provided an initial assessment of whether a decrease in resting HR as measured using smartphones in people’s natural settings could provide a sufficiently reliable indicator of abstinence to be used to verify self-reports.

The two most commonly used methods of verifying smoking abstinence are expired-air carbon monoxide (CO) concentration and cotinine concentration measured in saliva or urine.^1^ These can, in principle, be used in smoking cessation studies of remote interventions, where there is no face-to-face contact, but there are major challenges to doing so. In the case of CO, study participants must be sent a CO monitor and learn how to use it. The cost of the monitors and practical difficulties in getting people to use them mean that this approach remains problematic.^9,10^ Saliva cotinine has been used successfully but the costs are high when one takes into consideration the cost of the assay and incentivizing people to engage with what some see as an unattractive procedure in providing body fluids.^9^

Resting HR decreases by an average of around 5–15 beats per minute (bpm) within a day of stopping smoking and remains at that level for at least a year and probably indefinitely.^2–5^ This is most likely because of the acute effect of nicotine on the cardiovascular system, an effect that reverses as the concentration of nicotine in body tissues falls to zero.^11,12^ The nicotine-induced increase in heart rate is subject to acute tolerance, such that HR increases rapidly on ingestion of nicotine from the first cigarette of the day, but does not increase substantially as tissue nicotine concentrations change during the day.^13^ This effect appears to be reliable and it may occur with all, or almost all smokers.^11,14,15^ If that is the case then HR should decrease substantially on days when smoking has not occurred and should increase again if smoking does occur. This makes decrease in HR a potentially useful marker for verifying self-reports of smoking abstinence. At the same time, using nicotine replacement therapy (NRT) may or may not increase HR to the same extent as smoking. Thus, NRT use may confound HR measurements if these are used to verify smoking abstinence.

As a starting point in assessing the value of app-based HR measurement, it is important to determine whether a reliable decrease can be observed from smoking to abstinent days. Given the above considerations, it is also important to assess whether use of nicotine replacement products during abstinence reduces the size of the HR decrease.^16^

Thus, the following research questions were addressed:

1. Does resting HR, as assessed by a freely available smartphone app by participants in their natural settings decrease reliably on days when they are abstaining from smoking compared with days when they are smoking?
2. Is this the case even if they are using a nicotine replacement product?

## Methods

### Design

This study involved a single-factor repeated-measures design. The study was approved by a Research Ethics Committee at UCL (CEHP/2013/508 and CEHP/2016/556).

### Participants

The inclusion criteria for the study were: (1) ≥18 years old, (2) self-classified as healthy, (3) daily smoker of 5+ cigarettes per day, and (4) access to a suitable smartphone throughout the day. The recruitment was through posters, online, and by personal contact. Eighteen participants were recruited. Their mean age was 31.9 years (*SD* = 13.4); eight (44%) were female; their mean daily cigarette consumption was 12.2 (*SD* = 4.5) cigarettes per day. Participants used a single NRT product during their day of not smoking with NRT, and the patch was a common choice, but detailed information about NRT use was not recorded.

All participants provided written consent. They were paid £100 in cash or Amazon vouchers (according to their preference) for completing the experiment and up to £10 to pay for NRT products of their choice.

### Procedures

During the initial meeting with the researcher, participants were trained in the study procedures and downloaded one of two possible HR apps that met predefined criteria (not requiring registration, being free of charge, and having among the highest ratings on app stores): “Instant Heart Rate” or “Cardiio.” The participants were also provided with a personal CO monitor^9,17^ manufactured by Bedfont Scientific Ltd (see Supplementary Material 1). Participants were asked to choose 3 days when they would measure their HR (did not need to be consecutive days), all of which had to be within 10 days of providing consent. They were then assigned their sequence of experimental conditions as determined by a random number generator constrained to ensure an equal number of participants in each sequence. Participants were asked to record all HR and CO data on a study form (See Supplementary Material 2) and to send a photo of the completed form via e-mail or WhatsApp to the research team at the end of each study day.

### Experimental Conditions

All participants took part in three conditions on separate days: (1) smoking as usual, (2) not smoking, and (3) not smoking but using an NRT product of their choice. The sequence of the three conditions (eg, 1-2-3, 1-3-2, 2-1-3, etc.) was balanced across participants. Adherence to the requirements for abstinence in conditions (2) and (3) was assessed by self-measurement of expired-air CO concentrations. A reading of less than 10 parts per million was judged to confirm abstinence.

### Measures

On each of the three HR measurement days, participants were instructed to use the HR app to measure their HR (in bpm) five times at the following timepoints: (1) soon after waking (pre-9 am), (2) morning (10–12 pm), (3) lunchtime (12–3 pm), (4) afternoon (3–5 pm), and (5) evening (post-5 pm). Participants were instructed to remain seated for at least 5 minutes prior to each HR measurement. For each of the five measurement timepoints on a given day, they used the app to assess their resting HR by taking five consecutive readings and recorded them on the form.

The HR data for each of the five timepoints were averaged to give five measurements for each of the three conditions. As participants may not have smoked yet in the morning, the three postnoon readings were used to calculate the mean HR for each condition.

Participants were instructed in use of the expired-air CO monitors at the end of each condition day and recorded their CO readings on each of the three experimental days on a form provided.

### Analyses

The data analysis plan was uploaded on Open Science Framework before the data were analyzed (https://osf.io/8uy5p/). Means and 95% confidence intervals (CI) of mean postnoon HR were calculated for each of the three conditions. The differences between the three means were compared using the one-way analysis of variance (ANOVA) with repeated measures. Planned pairwise comparisons with Sidak correction were made between each of the conditions: (1) smoking as usual and not smoking with no nicotine product, (2) smoking as usual and not smoking but using a nicotine product, and (3) not smoking with no nicotine product and not smoking with a nicotine product. Given that HR data violated assumptions of normal distribution, in the sensitivity analysis we compared the means from the three conditions using Friedman’s test, which is a nonparametric equivalent test to repeated measures ANOVA.

## Results

Smoking abstinence during not smoking conditions without and with nicotine products was verified by CO in 18/18 (100%) and in 16/18 (88.9%) cases, respectively. Cases were analyzed on an intent to treat basis.

**Figure 1.**
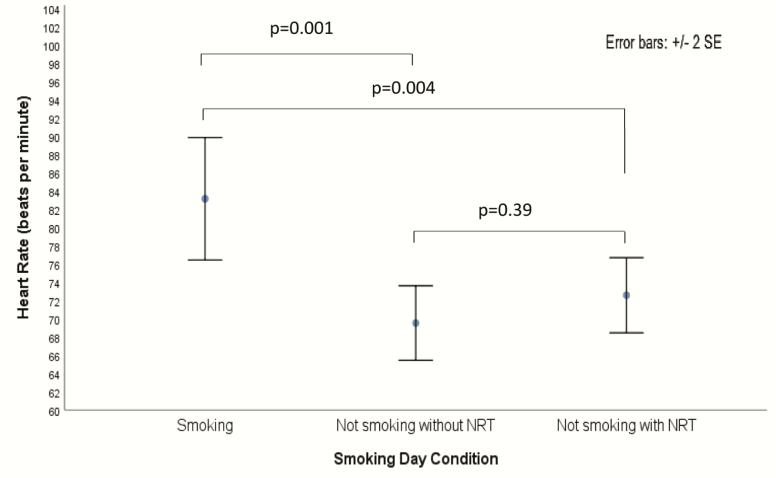
Mean heart rate (bpm) in the afternoon for three smoking conditions: smoking, not smoking without nicotine replacement therapy (NRT), not smoking with NRT.

A decrease in postnoon HR was observed in all cases from smoking as usual to not smoking without a nicotine product, and in 15 of 18 cases to not smoking with a nicotine product (see Supplementary Material 3). HR averaged 83.4 bpm (95% CI = 76.7 to 90.2) in the smoking as usual condition, 70.1 bpm (95% CI = 65.8 to 74.3) in the not smoking without NRT product condition, and 73.0 bpm (95% CI = 68.8 to 77.2) in the not smoking with NRT product condition. The smoking as usual condition had a statistically significant effect on HR (*F*(2, 34) = 14.37, *p* < .001; see Figure 1) when calculated using repeated-measure ANOVA. The results remained the same in the nonparametric Friedman test (*p* < .001). Compared with the smoking as usual condition, mean HR was 13.4 bpm lower (95% CI = 5.4 to 21.4, *p* = .001) in the not smoking without NRT condition and 10.4 bpm lower (95% CI = 3.1 to 17.8, *p* = .004) in the not smoking with NRT condition. There was no statistically significant difference in HR between the two not smoking conditions (*p* = .39).

## Discussion

Measurement of HR using a smartphone app showed a reliable decrease on a day of smoking abstinence compared with when smoking as usual. The decrease in HR on not smoking days but when a nicotine product was used was still substantial and significant, but may be less reliable. The magnitude of the observed decrease in resting HR was in line with previous findings.^2,4^ These results provide an initial indication that smartphone-assessed differences in HR may provide a basis for objective verification of smoking status.

The afternoon HR on a not smoking day with nicotine products was on average 2.9 bpm higher than on a not smoking day without NRT, in line with past literature.^15^ However, the difference was not significant. This could be due to low power. Alternatively, nicotine on its own may not be solely responsible for the increase in HR during smoking. Finally, this could also be explained by the different kinetics of nicotine absorption or lower nicotine intake from nicotine products in comparison to cigarettes. Future studies should explore the impact on HR of different intake levels of nicotine products during the not smoking days.

The next stage of the process of evaluating smartphone-assessed HR for verifying smoking cessation is to conduct a larger study to establish sensitivity and specificity at different thresholds of changes in HR for smokers who have maintained abstinence and who have smoked. This would allow a receiver operating characteristic (ROC) curve to be generated and a more definitive picture of the value of this method to emerge.^18^ It would also be important to collect data on the HR measuring app used by the participants, and to conduct a direct comparison of the readings from two or more of such apps. The time since last cigarette smoked before taking HR measurements should also be collected as a statistical control and to enable additional analyses of the latency effect between abstinence and changes in the HR.

If this method of verifying abstinence were to prove accurate, it would greatly improve our ability to assess abstinence on multiple occasions at a very low cost.

With the advent of wearable devices that automatically measure HR, the opportunity to use HR to assess and motivate abstinence may be considerably enhanced. It would in theory be possible to link such devices, for example, a Fitbit, to a smoking cessation app through an application program interface with no effort required from smokers themselves. Such devices have accelerometers and so it should be possible to ensure that HR is only recorded when users are resting.

## Conclusion

In this exploratory study, it was found that smoking abstinence significantly reduced HR assessed using smartphone apps in natural settings, even when a nicotine product was used. This could open up possibilities for use of remotely assessed HR to verify smoking abstinence in cessation studies.

### Supplementary Material

A Contributorship Form detailing each author’s specific involvement with this content, as well as any supplementary data, are available online at https://academic.oup.com/ntr.

## Supplementary Material

ntaa021_suppl_Supplementary_Materials

ntaa021_suppl_Supplementary_Taxonomy_Form
