# Supplementary material for: Decrease in Resting Heart Rate Measured Using Smartphone Apps to Verify Abstinence From Smoking: An Exploratory Study
Source: Nicotine Tob Res. 2020 Jan 23;22(8):1424–7. doi: 10.1093/ntr/ntaa021 (PMC7364830; doi:10.1093/ntr/ntaa021)
Supplement: ntaa021_suppl_Supplementary_Materials [file ntaa021_suppl_supplementary_materials.docx]

**Supplementary Materials**

**Supplementary Material 1**

**Box A1:** CO monitors used in this study

| As part of the HR study, participants were provided with a personal smartphone-based carbon monoxide (CO) monitor developed by Bedfont Scientific Ltd (called ‘Smokerlyzer’) to validate their self-reported abstinence on the non-smoking days with and without NRT. The smartphone-based monitor required a dedicated app to function. The app developed by the manufacturers, the Bedfont Scientific Ltd, was suitable for iOS and Android and was called ‘Smokerlyzer’. A second option for the Android users was ‘CO Monitor’ (developed at UCL) [[1](#_ENREF_1)]. The ‘CO Monitor’ app was preferred as it showed an exact reading in particles per million (ppm), and users of the ‘Smokerlyzer’ app occasionally reported technical problems with the app or connections between the monitor and the app.  In case of any technical difficulties with the smartphone-based Smokerlyzer monitor, participants were provided with an older and now discontinued model of personal CO monitors that connected to Windows PC (CompactUSB Smokerlyzer, also developed by Bedfont Scientific Ltd) [[2](#_ENREF_2)]. Participants using the PC-based CO monitors were asked to follow the CO testing schedule where possible, and to take at least one reading in the evening. |
| --- |

1. Herbec, A., Perski, O., Shahab, L., and West, R., *Smokers' Views on Personal Carbon Monoxide Monitors, Associated Apps, and Their Use: An Interview and Think-Aloud Study.* Int J Environ Res Public Health, 2018. **15**(2): p. E288.

2. Herbec, A., Brown, J., Shahab, L., and West, R., *Lessons learned from unsuccessful use of using personal carbon monoxide monitors to remotely assess abstinence in a pragmatic trial of a smartphone stop smoking app – A secondary analysis.* Addictive Behaviors Reports, 2018.

**Supplementary Material 2**

**Daily Diary form (Template for 3 versions, depending on Condition day)**

**Participant code:………………….**

**Heart rate and CO measurement record sheet**

Condition day:

**[1] Smoking as usual / [2] No smoking but use of NRT / [3] No smoking and no NRT**

Instructions: Each time you complete a reading, please enter it in the table below. If you want, you can type any comments in the box at the bottom of the page. Please send the completed sheet to <reseracher’s name> at the end of each day (via email: ….@ucl.ac.uk or WhatsApp: ……).

|  | On waking (pre-9am) | Morning  (10am-12pm) | Lunchtime  (12pm-3pm) | Afternoon  (3pm-5pm) | Evening  (post-5pm) |
| --- | --- | --- | --- | --- | --- |
| Heart rate (5 readings at each time point) | 1. | 1. | 1. | 1. | 1. |
|  | 2. | 2. | 2. | 2. | 2. |
|  | 3. | 3. | 3. | 3. | 3. |
|  | 4. | 4. | 4. | 4. | 4. |
|  | 5. | 5. | 5. | 5. | 5. |
| CO reading  (in ppm or parts per million) |  |  |  |  |  |
| Nicotine products used so far *[on the day when NRT will be used]* |  |  |  |  |  |
| Comments |  | | | | |

**Supplementary Material 3**

**Raw data, syntax and individual participant findings.**

**Table A1:** Raw data from the study. Wave signifies the wave of participant recruitment into the study.

|  |  | **Heart Rate (beat per minute)** | | | **Carbon monoxide levels** | | | |
| --- | --- | --- | --- | --- | --- | --- | --- | --- |
| Wave | **ID** | **Smoking** | **Non-smoking without NRT** | **Non-smoking with NRT** | **Smoking** | **Non-smoking without NRT** | **Non-smoking with NRT** |  |
| 1 | 1 | 94 | 77 | 78 | 22 | 3 | 4 |  |
| 1 | 2 | 71 | 60 | 63 | 10 | 3 | 2 |  |
| 1 | 3 | 88 | 83 | 75 | 21 | 7 | 11 |  |
| 1 | 4 | 73 | 68 | 65 | 21 | 6 | 14 |  |
| 1 | 5 | 88 | 76 | 73 | 18 | 2 | 4 |  |
| 1 | 6 | 84 | 60 | 66 | 15 | 5 | 5 |  |
| 2 | 7 | 61 | 59 | 82 | 18 | 3 | 9 |  |
| 2 | 8 | 123 | 63 | 84 | 26 | 1 | 0 |  |
| 2 | 9 | 86 | 72 | 78 | 14 | 2 | 7 |  |
| 2 | 10 | 83 | 73 | 71 | 9 | 3 | 2 |  |
| 2 | 11 | 80 | 66 | 61 | 11 | 2 | 2 |  |
| 2 | 12 | 90 | 85 | 90 | 29 | 3 | 3 |  |
| 2 | 13 | 94 | 81 | 81 | 13 | 0 | 0 |  |
| 2 | 14 | 79 | 69 | 64 | 10 | 2 | 2 |  |
| 2 | 15 | 69 | 62 | 71 | 8 | 3 | 2 |  |
| 2 | 16 | 81 | 63 | 68 | 13 | 8 | 8 |  |
| 2 | 17 | 70 | 65 | 64 | 8 | 3 | 3 |  |
| 2 | 18 | 88 | 79 | 80 | 12 | 3 | 3 |  |
| SPSS variable  name | | MeanSmok | MeanNoSmok | MeanNoSmokNRT | COSmok | COnoSmok | COnoSmokNRT |  |

Wave= we initially collected data from only six participants to assess feasibility, and then progressed with additional data collection.

**SPSS Syntax**

#Planned annalysis

GLM MeanSmok MeanNoSmok MeanNoSmokNRT

/WSFACTOR=factor1 3 Polynomial

/METHOD=SSTYPE(3)

/EMMEANS=TABLES(factor1) COMPARE ADJ(Sidak)

/PRINT=DESCRIPTIVE ETASQ OPOWER

/CRITERIA=ALPHA(.05)

/WSDESIGN=factor1.

#Sensitivity analysis using non-parametric test:

NPAR TESTS

/FRIEDMAN=MeanSmok MeanNoSmok MeanNoSmokNRT

/STATISTICS DESCRIPTIVES QUARTILES

/MISSING LISTWISE.

**Figure A1:** Mean heart rate in the afternoon for each participant on the three smoking condition: smoking (orange), non-smoking with NRT (blue), non-smoking without NRT (green).
